# Unveiling the Antibacterial Properties of Statins: An *In Vitro* Study on *Helicobacter pylori*

**DOI:** 10.1155/2024/6380155

**Published:** 2024-08-12

**Authors:** Manijeh Ebrahimzadeh, Fariba Asgharpour, Javad Shokri Shirvani, Sohrab Kazemi, Ali Akbar Moghadamnia

**Affiliations:** ^1^ Student Research Committee Health Research Institute Babol University of Medical Sciences, Babol, Iran; ^2^ Department of Laboratory Sciences Faculty of Para-Medicine Babol University of Medical Sciences, Babol, Iran; ^3^ Cancer Research Center Health Research Institute Babol University of Medical Sciences, Babol, Iran; ^4^ Cellular and Molecular Biology Research Center Health Research Institute Babol University of Medical Sciences, Babol, Iran; ^5^ Department of Pharmacology and Toxicology School of Medicine Babol University of Medical Sciences, Babol, Iran

## Abstract

**Background:**

*Helicobacter pylori* (*H. pylori*), a widespread bacterial pathogen, is associated with various gastrointestinal diseases, including gastric cancer. Statins, widely prescribed cholesterol-lowering agents, have demonstrated pleiotropic effects, including potential antimicrobial properties. This *in vitro* study investigated the direct antibacterial effects of three clinically approved statins, simvastatin, atorvastatin, and rosuvastatin, against *H. pylori* isolates.

**Methods:**

*H. pylori* strains were isolated from gastric biopsies of dyspeptic patients and identified by microbiological techniques. The minimum inhibitory concentrations (MICs) of statins were determined using the agar dilution method, and their antimicrobial activity was evaluated by the disc diffusion method using different concentrations of simvastatin, atorvastatin, rosuvastatin, tetracycline, and amoxicillin. Scanning electron microscopy (SEM) was employed to examine the morphology of *H. pylori* cells.

**Results:**

The minimum inhibitory concentration (MIC) values (*μ*g/mL) of atorvastatin, rosuvastatin, simvastatin, tetracycline, and amoxicillin against *H. pylori* were 240 ± 20, 450 ± 20, 460 ± 15, 155 ± 30, and 140 ± 20, respectively. In the disc diffusion assay, atorvastatin and rosuvastatin produced significantly larger inhibition zones compared to simvastatin at all concentrations tested (*p* < 0.05). The inhibition zone diameters (mm) increased with higher statin concentrations, ranging from 9 ± 1.4 to 13 ± 1.4 for atorvastatin, 8 ± 0.9 to 11 ± 0.6 for rosuvastatin, and 5 ± 1.3 to 6 ± 1.4 for simvastatin at the highest tested concentration (1200 *μ*g/ml). SEM analysis revealed the characteristic spiral morphology of *H. pylori* cells.

**Conclusion:**

Statins demonstrated varying degrees of antibacterial activity against *H. pylori* isolates, with atorvastatin exhibiting the highest potency. While the observed effects were lower than those of conventional antibiotics, these findings suggest the potential of statins as adjunctive agents or alternative therapeutic options, warranting further investigation through *in vivo* studies and clinical trials.

## 1. Introduction

*Helicobacter pylori* (*H. pylori*) is a spiral Gram-negative bacterium usually acquired in childhood through the orofecal route. Without therapeutic eradication, *H. pylori* can persist in the human stomach for an entire lifetime [1, 2]. *H. pylori* infection is known to influence more than 50% of the worldwide population. The pathogen is assumed to be associated with an increased risk of gastrointestinal illnesses, including peptic ulcers, gastritis, and mucosa-associated lymphoid tissue (MALT) lymphoma. *H. pylori* infection also results in chronic oxidative stress, which eventually may lead to the development of gastric adenocarcinoma [3, 4]. The most important cause of stomach cancer, which is known as the third cause of cancer-related death, can be considered *H. pylori* infection, whose eradication alone can reduce the risk of stomach cancer by 47% [5]. Besides, several studies have demonstrated a link between *H. pylori* infection and extragastric diseases such as vitamin B12 deficiency anemia, iron deficiency anemia, primary immune thrombocytopenia (PIT), Parkinson's disease, psoriasis, and Guillain–Barrè syndrome [6, 7]. *H. pylori* eradication therapies include triple or even quadruple antibiotic-based regimens. Despite the effort, eradication failure has increased due to escalating antimicrobial resistance of *H. pylori*, which has reached alarming rates worldwide [8, 9]. Also, some factors, such as more acidic gastric juice and being overweight, can contribute to a lower eradication rate [10, 11]. In addition to antimicrobial resistance, antibiotic overuse may cause unintended consequences such as short-term impairment of the gut normal flora and increased risk of asthma and allergy [12].

Statins inhibit 3-hydroxy-3-methylglutaryl coenzyme A (HMG-CoA) reductase and were discovered in the 1970s [13]. They are among the most commonly prescribed medications due to their cholesterol-lowering effects. In the guidelines, they have been suggested as a frontline treatment for primary and secondary prevention of cardiovascular diseases [14, 15]. In addition, statins have been demonstrated to have some cholesterol-independent or pleiotropic impacts, including anti-inflammatory, anticancer, and immunomodulatory functions [16]. Studies have demonstrated that statins have a positive effect on reducing the risk of severe bacterial infections, such as *Chlamydia pneumoniae*, *Clostridium difficile*, *Staphylococcus aureus*, and *Streptococcus pneumoniae* infections [17–19]. In addition, various studies have reported that statins can improve the outcome of bacterial infections such as wound infections, pneumonia, and sepsis [20–24]. Clinical trials have investigated adding statins in *H. pylori* eradication regimen, and the results have shown the effectiveness of different statins in *H. pylori* eradication [25–27]. Furthermore, there is growing evidence suggesting that statins have a direct inhibitory effect on the *in vitro* growth and virulence of different bacterial pathogens [28, 29].

The emergence of multidrug-resistant bacteria, such as new *H. pylori* strains, *Klebsiella pneumoniae*, *Staphylococcus aureus*, and *Pseudomonas aeruginosa* compromises our ability to treat common infections [30, 31]. As a result, there is a need to develop new strategies to address the issue of antimicrobial resistance. One strategy is repurposing existing commercial drugs, such as statins, as a therapeutic alternative to antimicrobial resistance [32]. Although statins have been shown to cause myopathy and type 2 diabetes mellitus, these are the only reliably proven adverse effects. However, some studies suggest that statin use may also increase the risk of hemorrhagic stroke, cataracts, cognitive impairment, and liver injury [33–35].

In this study, we investigated the direct antibacterial effect of three clinically approved statins, including simvastatin (SMV), atorvastatin (ATV), and rosuvastatin (RSV) against *H. pylori*, one of the most common pathogens in the world.

## 2. Materials and Methods

### 2.1. Study Design and Participants

This controlled, multicentric study was conducted from March 2020 to January 2022 at Babol University of Medical Sciences, Iran. The study protocol was approved by the Research Ethics Committee of Babol University of Medical Sciences (IR.MUBABOL.REC.1400.232), and all participants provided written informed consent.

A total of 40 patients with dyspeptic symptoms were recruited from the endoscopy units of Shahid Beheshti and Shahid Yahyanejad hospitals in Babol, Iran. Gastric antral and corporal biopsies were obtained from these patients during upper gastrointestinal endoscopy, and clinical diagnoses were made based on endoscopic findings by experienced gastroenterologists. The inclusion criteria were patients with dyspeptic symptoms undergoing upper gastrointestinal endoscopy. Exclusion criteria were as follows: (1) use of proton pump inhibitors (PPIs), histamine type 2 receptor antagonists (H2RAs), bismuth salts, or antibiotics within the preceding two weeks; (2) presence of malignancies; and (3) contraindications for upper gastrointestinal endoscopy.

### 2.2. Isolation and Identification of *H. pylori*

Gastric biopsy samples were transported to the microbiology laboratory at the School of Allied Sciences, Babol University of Medical Sciences, in 9% saline solution at 4°C within 2 hours of collection. Biopsies were homogenized, and a portion was used for the urease test, while the remaining tissue was cultured for *H. pylori* isolation. For culture, homogenized samples were inoculated onto selective Brucella agar plates (Merck, Germany) supplemented with 7% (v/v) defibrinated sheep blood, 10 mg/L vancomycin, 5 mg/L trimethoprim, and 2.5 mg/L amphotericin B. Plates were incubated at 37°C for 3–7 days under microaerobic conditions (80–90% N_2_, 5–10% CO_2_, and 5–10% O_2_) using Gas Pack C (Anaerocult C, Merck) in an anaerobic jar. After 3 days of incubation, plates were examined for the presence of circular, smooth, and translucent *H. pylori* colonies (0.5–1 mm diameter). Presumptive identification of *H. pylori* was based on colony morphology, gram staining (curved or straight Gram-negative rods), and positive results for catalase, oxidase, and urease tests. Plates with no visible growth were incubated for up to 7 days before being discarded as negative.

### 2.3. Scanning Electron Microscopy (SEM)

For SEM analysis, *H. pylori* bacterial suspension was prepared in Brucella blood broth medium at a turbidity equivalent to 0.5 McFarland standard and cultured at 37°C. The bacterial cells were washed with phosphate-buffered saline (PBS) and fixed with 3% glutaraldehyde solution for 2 hours at room temperature. After fixation, the cells were washed again with PBS and dehydrated using a graded ethanol series (50%, 70%, 90%, and 100%). The dehydrated specimens were air-dried for 24 hours. Prior to SEM examination, the dried bacterial samples were sputter-coated with gold-palladium to enhance conductivity. The surface morphology of *H. pylori* cells was then investigated using a scanning electron microscope (SNE-4500M, SEC CO., LTD, Suwon, Korea) [36, 37].

### 2.4. Antimicrobial Susceptibility Testing

The antimicrobial susceptibility of *H. pylori* isolates was evaluated using agar dilution and disc diffusion methods, following the guidelines of the Clinical and Laboratory Standards Institute (CLSI) [38].

#### 2.4.1. Agar Dilution Method

Brucella agar plates supplemented with 7% (v/v) defibrinated sheep blood were prepared with two-fold serial decreasing concentrations (15, 30, 60, 120, 240, and 480 *μ*g/mL) of SMV, ATV, and RSV. Bacterial suspensions were prepared from fresh subcultures in saline and adjusted to a turbidity equivalent to 0.5 McFarland standard (approximately 1.5 × 10^8^ CFU/mL). Each plate was inoculated with 10 *μ*L of the bacterial suspension and incubated under microaerobic conditions at 37°C for three days, as described earlier. The minimum inhibitory concentration (MIC) was defined as the lowest concentration of the antimicrobial agent that completely inhibited visible bacterial growth. Tetracycline (TET) and amoxicillin (AMOX) at concentrations of 40, 80, 160, and 320 *μ*g/mL, as well as a drug-free sterile medium, were included as controls.

#### 2.4.2. Disc Diffusion Method

The antibacterial activity of SMV, ATV, and RSV was evaluated with the Kirby–Bauer disc diffusion method. Sterile blank discs (6 mm) were inoculated with 10 *μ*l of 400, 800, and 1200 *μ*g/ml of SMV, ATV, and RSV (400, 800, and 1200 *μ*g/ml). AMOX and TET (40, 80, 160, and 320 *μ*g/ml) and the no drug/solvent discs served as controls. For each case, 10 *μ*l of 0.5 McFarland bacterial suspension was spread on Brucella blood agar's Petri dish. Discs were placed with sterilized forceps at a distance of 24 mm on a dry agar surface. After incubation for three days under microaerobic conditions, the diameter of the inhibition zones around each disc was measured using a metric ruler.

### 2.5. Statistical Analysis

Data analyses were performed using SPSS software version 22.0 (IBM, Armonk, NY, USA). The Shapiro–Wilk test was employed to assess the normality of the numerical data distribution. For comparisons of mean values, parametric tests (one-way analysis of variance (ANOVA)) and nonparametric tests (Kruskal–Wallis and Mann–Whitney) were utilized as appropriate. A *p* value of less than 0.05 was considered statistically significant.

## 3. Results

### 3.1. Patient Characteristics

The study included a total of 40 patients with a mean age of 52.85 years (range: 27–82 years). The study comprised 23 males (57.5%) and 17 females (42.5%). Endoscopic evaluation revealed gastritis as the most prevalent finding, observed in 25 patients (62.5%), followed by peptic ulcers in 15 patients (37.5%). A total of 65 tissue samples were obtained from these patients. The demographic characteristics and endoscopic findings of the study participants are summarized in Table 1. Of the 65 biopsy samples analyzed, 35 (53.84%) were positive for the urease test, indicating the presence of active *H. pylori* infection. However, only 10 samples (15.38%) yielded positive cultures for *H. pylori*.

### 3.2. Isolation and Identification of *H. pylori*

**Figure 1:**
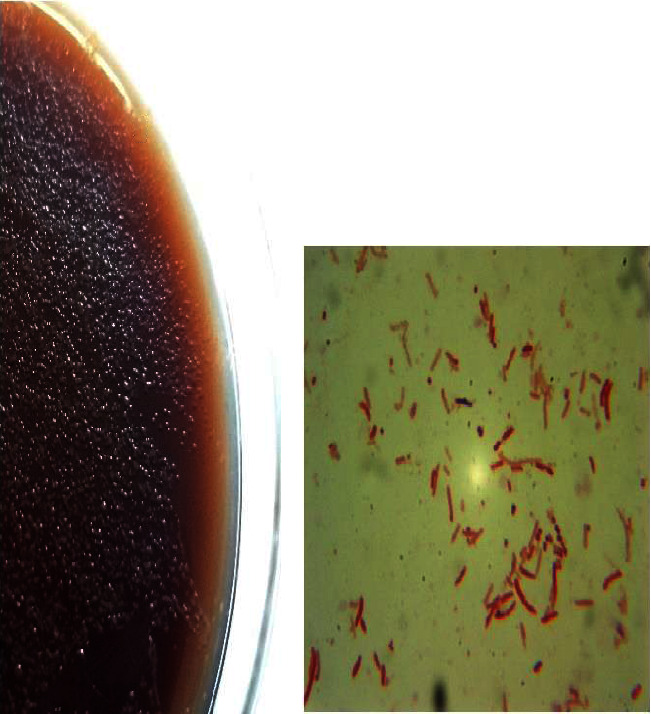
Isolation and identification of *Helicobacter pylori*. (a) Typical appearance of *H. pylori* colonies on selective Brucella agar plates. The colonies exhibit a circular, smooth, and translucent morphology with diameters of 0.5–1 mm. (b) Gram staining of the isolated *H. pylori* bacteria, showing the characteristic curved or spiral-shaped, Gram-negative rods.

**Figure 2:**
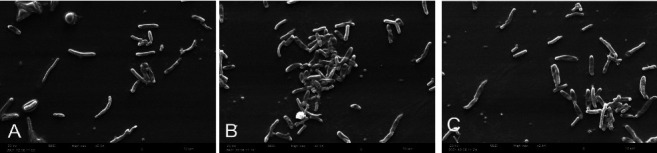
Scanning electron micrograph depicting the characteristic spiral morphology of *H. pylori* bacterial cells isolated from gastric biopsy samples. The bacteria in groups A (AMOX), B (SMV), and C (ATV) were cultured, fixed, dehydrated, and sputter-coated with gold-palladium before imaging by scanning electron microscopy.

Among the 65 biopsy samples analyzed, 35 (53.84%) tested positive for the urease test, indicating the presence of *H. pylori* infection. Subsequent culture isolation efforts yielded 10 (15.38%) positive cultures for *H. pylori.* SEM analysis of the cultured isolates revealed the characteristic morphology of *H. pylori* bacteria, which is a spiral-shaped rod (Figures 1 and 2).

### 3.3. Antibacterial Assay

**Figure 3:**
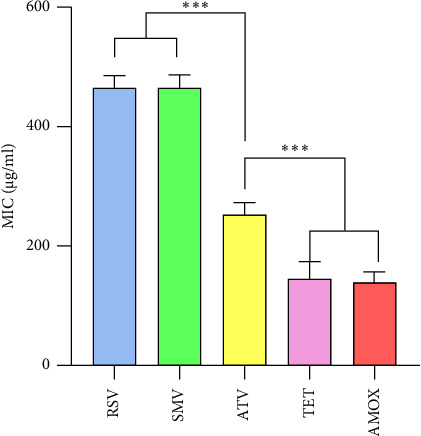
The minimum inhibitory concentration (*μ*g/ml) against *H. pylori*: the MICs of tetracycline and amoxicillin are included for comparison. Data are presented as mean ± standard deviation. ^*∗∗∗*^*p* < 0.001 compared to atorvastatin (*n* = 10 sample).

The MICs of the tested antimicrobial agents against *H. pylori* isolates were determined by the agar dilution method. ATV exhibited the lowest MIC (240 ± 20 *μ*g/mL) among the statins tested, followed by RSV (450 ± 20 *μ*g/mL) and SMV (460 ± 15 *μ*g/mL). The MIC values for the control antibiotics, TET and AMOX, were 155 ± 30 *μ*g/mL and 140 ± 20 *μ*g/mL, respectively. ATV demonstrated significantly lower MIC values compared to SMV and RSV (*p* < 0.001). In addition, TET and AMOX exhibited significantly lower MIC values than all three statins (*p* < 0.001).  Figure 3 shows the average MICs attained for ATV, SMV, and RSV against *H. pylori*. The sterile medium without any drug did not interfere with bacterial growth.

The disc diffusion method was employed to evaluate the antimicrobial activity of the statins by measuring the diameter of the inhibition zones produced against *H. pylori*. As shown in Table 2, increasing concentrations of ATV, RSV, and SMV (400, 800, and 1200 *μ*g/ml) resulted in larger inhibition zones, ranging from 5 ± 1.3 mm to 13 ± 1.4 mm. ATV and RSV exhibited significantly larger inhibition zones compared to SMV at all tested concentrations (*p* < 0.05). Larger inhibition zones of AMOX at concentrations less than 320 *μ*g/ml were not significantly different from ATV 800 and 400 *μ*g/ml and RSV 400 *μ*g/ml. TET showed significantly larger inhibition zones compared to SMV at all tested concentrations (*p* < 0.05). The bacterial growth inhibition zone is raised with increasing statin concentrations.

## 4. Discussion

The escalating rates of antibiotic resistance and the drugs' adverse effects indicate a rising need to find new ways to face infections with fewer adverse events and higher or similar beneficial properties. A part of statins' favorable role in infectious diseases may arise from their antimicrobial properties. In our study, all three statins showed some *in vitro* antibacterial activity against *H. pylori*. ATV was the most potent, followed by RSV and SMV, respectively. RSV achieved better MIC values and inhibition zone diameter than SMV. Results of the current study demonstrate that the antibacterial effect increases dose-dependently.

To the best of our knowledge, no previous studies have investigated the *in vitro* antibacterial effect of statins on *H. pylori*. However, some reports have demonstrated that statins have a direct antibacterial effect against other bacterial strains. The MIC values reported against different pathogens were inconsistent and ranged from 15 to 500 *μ*g/ml [24, 28, 29, 39–43]. According to a study conducted by Masadeh et al., it was found that ATV and SMV were more effective in inhibiting the growth of bacterial strains in lower MICs compared to RSV. These results were consistent with our findings. In contrast, the same values for ATV (108.33 and 216.67 *μ*g/ml) and SMV (116.67 and 291.67 *μ*g/ml) were significantly lower. We utilized the same agar medium supplemented with drugs as the study mentioned earlier; however, different bacterial isolates exhibited varying degrees of susceptibility to the same antimicrobial drugs [29]. The emergence of multidrug-resistant strains of *H. pylori*, *Klebsiella pneumoniae*, *Staphylococcus aureus*, and *Pseudomonas aeruginosa* restricts our ability to treat common infections. None of the tested statins was found to have any practical antibacterial effects against *Escherichia coli*, *Pseudomonas aeruginosa*, or *Serratia marcescens*, at least in tested concentrations [42]. In a study, RSV and ATV were observed to inhibit the growth of methicillin-susceptible and methicillin-resistant *S. aureus*, *P. aeruginosa*, *E. coli*, vancomycin-sensitive *E. faecalis*, and vancomycin-resistant *E. faecalis* strains at 100 and 250 *μ*g/ml concentrations, respectively. In this study, RSV was more potent in inhibiting bacterial growth than ATV, which was in contrast with our results [43]. Statins inhibit HMG-CoA reductase, the enzyme necessary for the biosynthesis of isoprenes in bacterial cells. However, in prokaryotes, this enzyme is of a different structural class and has a 10,000 times lower affinity for statins; thus, the antibacterial effect of statins is unlikely to be related to the suppression of HMG-CoA reductase [44]. Statins are also known for pleiotropic effects such as cytotoxicity and apoptosis-promoting effects and suppress cell growth. The antimicrobial action of statins might be related to these properties [45–47]. Clinical trials have been conducted to assess the impact of adding ATV and SMV to *H. pylori* eradication therapy based on their antimicrobial and pleiotropic effect, and they observed improved eradication rates in experiment groups [25–27, 48]. Some researchers demonstrated the MIC value of around 30 *μ*g/ml for SMV against *S. aureus* strains [24, 40]. At the same time, other studies noted 64 and 75 *μ*g/ml as MIC values for SMV against the same strains [28, 42]. Using different solvents and culture media might be the reason for this conflict.

The mechanism of the antibacterial properties of statins still needs elucidating. SMV, pravastatin (PRV), and lovastatin (LVS) are type 1 statins that are considered to have fungal origins. According to prior studies, unlike PRV and LVS, SMV exhibited antibacterial effects [24]. ATV and RSV are classified as type 2 statins, which are synthetic compounds, and both have shown antibacterial activity [29]. Hence, the fungal origin might not be related to statins' antibacterial properties. The presence of two methyl groups with a tetrahedral or similar trigonal pyramidal in molecular geometry, evident in statins with antibacterial activity (ATV, RSV, SMV, and fluvastatin), has been hypothesized as a critical mechanism of the statin's antibacterial properties [49]. The mentioned structure can influence the bacteria in different ways, such as hydrogen bonds with lipopolysaccharide structures leading to Gram-negative bacterial surface breakdown, disruption in cellular controlling functions through nonpolar forces between statins and teichoic acids, and lipoteichoic acids in the surface of the Gram-positive bacteria. Lastly, the potential van der Waals forces and hydrogen bonds with surface proteins in other Gram-negative and Gram-positive bacteria interfere with surface consistency [49, 50]. In a study, 100 *μ*M of statins (SMV, LVS, and mevastatin) could decrease the swarming motility of *P. aeruginosa* in different strains [41]. It has been elucidated that bacteria motility is intrinsically related to other essential traits of bacterial virulence phenotypes, such as quorum sensing [51] and the ability to form a biofilm [52].

In the present study, the inhibition zones produced by statins were not more extensive than those of current antibiotics, and our reported MICs were higher than those in some previous studies. This discrepancy may be attributed to the emergence of new *H. pylori*-resistant strains, failed *H. pylori* eradication therapy, uncontrolled use of empirical antibiotic therapy for treating respiratory, genital, and urinary infections, as well as potential variations due to race and ethnicity [9, 53]. In addition, the *H. pylori* bacteria investigated in this study were retrieved from gastric biopsies, and clinical isolates have been observed to be less susceptible to antimicrobial agents compared to standard isolates [29].

We anticipated difficulties in receiving *H. pylori* eradication therapy due to the drug's side effects, such as diarrhea, taste disturbance, nausea, and abdominal pain [54]. Statins are a potential option for *H. pylori* eradication clinical trials to decrease the current drug dosage and treatment duration. However, our study has some limitations. The polymerase chain reaction method could have aided in identifying specific resistant strains and clarifying the effects of statins on the most frequent local strains. Furthermore, using standard-type isolates of various pathogens and clinical isolates could have improved our understanding of the antibacterial properties of statins.

## 5. Conclusion

This study demonstrated that statins exhibit antibacterial activity against *Helicobacter pylori* isolates, with atorvastatin showing the highest potency among the tested agents. These findings suggest that adding statins (especially atorvastatin) as a combination therapy for *H. pylori* is beneficial, particularly in the context of antibiotic resistance. Further research is warranted to elucidate the mechanisms underlying the antibacterial effects of statins and explore their synergistic potential with other antimicrobial agents.

## Figures and Tables

**Table 1:** Demographic characteristics of the patients and endoscopic findings.

	Descriptive statistics
(1) Mean age (years)	52.85 (27–82)
(2) Sex	
Male	23 (57.5%)
Female	17 (42.5%)
(3) Endoscopic findings	
Gastritis	25 (62.5%)
Peptic ulcer	15 (37.5%)

**Table 2:** The inhibition zone diameter (mm) of different components (*μ*g/ml) against *H. pylori*.

Drugs	*μ*g/ml						
	400	800	1200	40	80	160	320
ATV	9 ± 1.4	11 ± 0.9	13 ± 1.4	—	—	—	—
RSV	8 ± 0.9	10 ± 1.4	11 ± 0.6	—	—	—	—
SMV	5 ± 1.3	5 ± 1.1	6 ± 1.4	—	—	—	—
Tetracycline	—	—	—	10 ± 1.4	10 ± 1.8	11 ± 1.1	12 ± 0.6
Amoxicillin	—	—	—	10 ± 1.3	10 ± 1.1	12 ± 1.3	13 ± 0.6

## Data Availability

The data used to support the findings of this study are available from the corresponding author upon request.
